# Publisher Correction: Measuring the α-particle charge radius with muonic helium-4 ions

**DOI:** 10.1038/s41586-021-03360-2

**Published:** 2021-02-27

**Authors:** Julian J. Krauth, Karsten Schuhmann, Marwan Abdou Ahmed, Fernando D. Amaro, Pedro Amaro, François Biraben, Tzu-Ling Chen, Daniel S. Covita, Andreas J. Dax, Marc Diepold, Luis M. P. Fernandes, Beatrice Franke, Sandrine Galtier, Andrea L. Gouvea, Johannes Götzfried, Thomas Graf, Theodor W. Hänsch, Jens Hartmann, Malte Hildebrandt, Paul Indelicato, Lucile Julien, Klaus Kirch, Andreas Knecht, Yi-Wei Liu, Jorge Machado, Cristina M. B. Monteiro, Françoise Mulhauser, Boris Naar, Tobias Nebel, François Nez, Joaquim M. F. dos Santos, José Paulo Santos, Csilla I. Szabo, David Taqqu, João F. C. A. Veloso, Jan Vogelsang, Andreas Voss, Birgit Weichelt, Randolf Pohl, Aldo Antognini, Franz Kottmann

**Affiliations:** 1 Max Planck Institute of Quantum Optics, Garching, Germany; 2 QUANTUM, Institut für Physik & Exzellenzcluster PRISMA, Johannes Gutenberg-Universität Mainz, Mainz, Germany; 3 Institute for Particle Physics and Astrophysics, ETH Zurich, Zurich, Switzerland; 4 Paul Scherrer Institute, Villigen, Switzerland; 5 Institut für Strahlwerkzeuge, Universität Stuttgart, Stuttgart, Germany; 6 LIBPhys-UC, Department of Physics, University of Coimbra, Coimbra, Portugal; 7 Laboratory for Instrumentation, Biomedical Engineering and Radiation Physics (LIBPhys-UNL), Department of Physics, NOVA School of Science and Technology, NOVA University Lisbon, Caparica, Portugal; 8 Laboratoire Kastler Brossel, Sorbonne Université, CNRS, ENS-PSL Research University, Collège de France, Paris, France; 9 Physics Department, National Tsing Hua University, Hsincho, Taiwan; 10 i3N, Universidade de Aveiro, Aveiro, Portugal; 11 Ludwig-Maximilians-Universität, Fakultät für Physik, Munich, Germany; 12 Present Address: LaserLaB, Department of Physics and Astronomy, Vrije Universiteit, Amsterdam, The Netherlands; 13 Present Address: TRIUMF, Vancouver, British Columbia Canada; 14 Present Address: Institut Lumière Matière, University of Lyon, Université Claude Bernard Lyon 1, CNRS, Villeurbanne, France; 15 Present Address: Theiss Research, La Jolla, CA USA; 16 Present Address: Department of Physics, Lund University, Lund, Sweden

**Keywords:** Exotic atoms and molecules, Experimental nuclear physics

Correction to: *Nature* 10.1038/s41586-021-03183-1Published online 27 January 2021

This Article was originally published online on 27 January 2021 without Open Access. With the author(s)’ decision to opt for Open Access, the copyright of the Article changed on 17 February 2021 to © The Author(s) 2021, and the Article is now licensed/distributed under the terms of a Creative Commons Attribution 4.0 International License (http://creativecommons.org/licenses/by/4.0/.), which permits use, sharing, adaptation, distribution and reproduction in any medium or format, as long as you give appropriate credit to the original author(s) and the source, provide a link to the Creative Commons license, and indicate if changes were made. The original Article has been corrected online.

